# Complete Genome Sequence of *Trueperella pyogenes*, Isolated from Infected Farmland Goats

**DOI:** 10.1128/genomeA.01421-16

**Published:** 2016-12-15

**Authors:** Su-hui Zhang, Jin-jie Qiu, Rui Yang, Ke-fei Shen, Guo-yang Xu, Li-zhi Fu

**Affiliations:** a Chongqing Academy of Animal Sciences, Chongqing, People’s Republic of China; b Chongqing Engineering Research Center for Goat, Chongqing, People’s Republic of China

## Abstract

*Trueperella pyogenes* is a significant pathogen of livestock, causing diverse diseases, such as mastitis, liver abscessation, and pneumonia. In this study, we have reported the genome sequence of *Trueperella pyogenes* 2012CQ-ZSH. Moreover, several genes coding for virulence factors were found, such as pyolysin (PYO), *nanH*, *nanP*, *cbpA*, *fimC*, and *fimE*.

## GENOME ANNOUNCEMENT

*Trueperella pyogenes*, first named *Aeranobacterium pyogenes*, was reclassified into the genus *Trueperella* based on the phylogenetic analysis, according to Nagib et al. (1). *T. pyogenes* is a commensal and opportunistic pathogen of livestock, causing diverse diseases, such as mastitis, liver abscessation, and pneumonia. *T. pyogenes* expresses several main virulence factors that help it to infect diverse animals, including pyolysin (PLO), *nanH*, *nanP*, *fimA*, and *cpbA* (2). The expression of variety virulence factors may explain why *T. pyogenes* is able to cause such a diverse range of diseases.

*T. pyogenes* strain 2012CQ-ZSH is Gram positive, nonmotile, nonsporulating, and polymorphic. In this study, strain 2012CQ-ZSH was isolated from the infected goats from Chongqing, China, and its optimal temperature is 37°C. The genomic DNA of strain 2012CQ_ZSH was extracted using the DNeasy blood and tissue kit (Qiagen, Germany), according to the manufacturer’s protocol. The concentration and purity of DNA were measured by a NanoDrop spectrophotometer (ND1000; Thermo Fisher Scientific, DE). Genome sequencing of *T. pyogenes* strain 2012CQ-ZSH was performed at Sangon Biotech (Shanghai, China) employing the Illumina HiSeq 2500 sequencing platform. The clean data were used for assembly using Velvet 1.2.10 (3) and SPAdes 3.1.1 (4). The prediction of open reading frames (ORFs) was performed with Glimmer version 3.02 (5). Ribosomal RNAs were obtained by using RNAmmer version 1.2 (6), and tRNAs were identified by tRNAscan-SE version 1.21 (7). Then, the predicted genes were compared to NCBI nr, Swiss-Prot, Pfam, and COG databases. Meanwhile, we assigned translated amino acids into KEGG pathways using KEGG Automatic Annotation Server (KAAS) (8). SignalIP version 4.1 was used to identify genes with signal peptides, and THMMER 2.0 was performed to define genes with transmembrane helices (9). Clustered regularly interspaced short palindromic repeats (CRISPRs) were predicted by CRISPRfinder (10, 11).

The genome sequence is 2,295,822 bp, with a G+C content 59.70%. The whole-genome sequence contains 2,019 predicted genes, 46 tRNAs, and six rRNAs. The genome encodes several virulence factors, including PLO, *cbpA*, *nanH*, *nanP*, *fimC*, and *fimE*. Moreover, genes regulating the formation of biofilms were also found in the genome sequence, such as *luxS*. The presence of the genome sequence of *T. pyogenes* may provide new approaches to develop antimicrobial drugs different from the traditional drugs, such as antipathogenic drugs which will not lead to resistant strains (12).

In conclusion, the complete genome sequence of *T. pyogenes* will provide us new insights into its pathogenesis and virulence power. More importantly, the detailed information of the genome sequence offers a systematic approach for choosing vaccines and protecting local livestock from the infections.

### Accession number(s).

This whole-genome shotgun project has been deposited in DDBJ/EMBL/GenBank under the GenBank accession no. CP012649. The version described in this paper is the first version, CP012649.1.
